# Measles containing vaccine coverage and factors associated with its uptake among children aged 24–59 months in Cherangany Sub County, Trans Nzoia County, Kenya

**DOI:** 10.1371/journal.pone.0263780

**Published:** 2022-02-23

**Authors:** Stella Mamuti, Collins Tabu, Irene Marete, Davies Opili, Rose Jalang’o, Ahmed Abade

**Affiliations:** 1 Kenya Field Epidemiology and Laboratory Training Program, Ministry of Health, Nairobi, Kenya; 2 Department of Health, Trans Nzoia County, Kitale, Kenya; 3 National Vaccine and Immunization Program, Ministry of Health, Nairobi, Kenya; 4 Department of Child Health and Pediatrics, Moi University, Eldoret, Kenya; Illawarra Shoalhaven Local Health District, AUSTRALIA

## Abstract

**Introduction:**

Measles is a vaccine-preventable disease whose elimination depends on the measles-containing vaccine (MCV) coverage of ≥95% in the population. In 2020, Kenya reported 597 cases, an increase of 158 cases from those reported in 2019. This study aimed to estimate the measles vaccine coverage and factors associated with its uptake in Cherangany Sub County.

**Methods:**

We conducted a cross-sectional study using cluster sampling in the Cherangany Sub County of Trans Nzoia County in May 2021. We enrolled eligible children aged between 24–59 months and interviewed their caregivers using a structured questionnaire. We conducted descriptive, bivariate, and multivariate analyses. We used Prevalence Odds Ratio (POR) at bivariate and adjusted POR (aPOR) at multivariate with their corresponding 95% confidence interval as the measure of association. We regarded the variables with a p-value of less <0.05 at the multivariate level as independently associated with immunization status.

**Results:**

We recruited 536 eligible children. The median age of the participants was 39 months (Interquartile Range 31–50). The coverage was 96.6% (518/536) for MCV dose one (MCV 1), and 56.2% (301/536) MCV dose two (MCV 2). At the bivariate level, family monthly income (POR 2.32, 95% CI 1.14–4.72), child vaccination status for other scheduled vaccines (POR 0.21, 95% CI 0.07–0.66), caregiver’s level of education (POR = 1.82, 95% CI 1.29–2.57), knowledge of the vaccine-preventable diseases (POR = 0.55, 95% CI 0.38–0.80), and knowledge of the number of MCV scheduled doses (POR = 0.13, 95% CI 0.09–0.02) were significantly associated with MCV uptake. The Caregiver’s knowledge on the number of MCV scheduled doses (POR = 5.73, 95% CI 3.48–9.45) and children whose birth order was ≤5^th^ born (POR = 0.5, 95% CI 0.22–0.95) were significantly associated with MCV uptake at the multivariate analysis.

**Conclusion:**

The MCV 2 coverage was lower than the WHO recommended ≥ 95%. Lack of knowledge of the number of MCV scheduled doses and the child’s birth order in the family are factors associated with not being fully vaccinated against measles.

**Recommendation:**

There is a need to strengthen the defaulter tracing system to follow up the children who default after receiving MCV 1, focusing interventions on the identified factors.

## Introduction

Measles, a vaccine-preventable disease, continues to be an important public health problem globally. In 2020, 149,796 measles cases were reported globally, with the African region contributing 115,364 (77%). Kenya reported 597 cases in the same year, an increase of 158 cases from those reported in 2019 [1]. To reduce the burden of measles, the World Health Organization (WHO) recommends coverage of ≥ 95% of two doses of Measles Containing Vaccine (MCV) to assure immune response and interrupt the transmission. Kenya offers two doses of the Measles-Rubella (MR) vaccine to children; the first dose at nine months and the second at 18 months old. The vaccine is provided at no cost at either the health facilities or outreach sites in areas considered hard to reach, such as areas with a distance of more than 5 kilometers (Km) to the nearest health facility. The country also carries out supplemental MCV immunization activities such as campaigns and intensified routine immunization for children ages five years and below to improve the coverage.

In 2019, the administrative data coverage for children under one-year-old for MCV 1 and 2 in Kenya was 89% and 45%, respectively. The Trans Nzoia County reported 69% for MCV 1 and 24% for MCV 2 coverages. The Cherangany Sub County had 52.4% and 22.1% MCV 1 and 2 coverages, respectively, from the administrative data, the lowest in the county for the same year [2].

Most research focused on children ages 12 to 23 months which does not include the MCV dose 2. In Kenya, related research focused on special populations such as the nomadic, the urban poor, and immigrants and asylum seekers. The Kenya Health Survey, 2014 only described several factors associated with vaccination uptake, including the maternal level of education, family wealth index, and birth order [3]. However, the strength of association was not identified; thus, the findings could not be extrapolated to the whole country. This study sought to determine the MCV coverage and factors associated with its uptake among children ages 24 to 59 months in Cherangany Sub County in Trans Nzoia County, Kenya, 2021.

## Methods

### Study design and setting

We conducted a cross-sectional study in Cherangany Sub County in May 2021. The Sub- County is in Trans Nzoia County, which is in the North rift region of Kenya. The Sub County has a population of 282,560 with a population density of 448.7 population/km^2^, seven wards (the lowest administrative unit that elects the county assembly member), and 66 health facilities. The top five diseases among the pediatrics in the county include respiratory tract infections, malaria, diarrhea, skin diseases, and urinary tract infections. The county average vaccination coverage is 55%, below the national target of 80%. The average hospital delivery is 42% despite having 92% of the mothers attending the 1st Antenatal Care (ANC) visit. The median period between two live births in the county is 35.5 months. This is comparable to the whole of Kenya (median 36.3 months) [4].

### Study population and sampling procedure

#### Study population and period

We enrolled children ages 24 to 59 months residing in the sub-county in May 2021.

#### Sample size calculation

The vaccination cluster survey formula recommended by WHO was adopted in this study [5]. The MCV 2 coverage for the Sub- County (p) = 22%. We calculated the sample size based on the following formula:

N minimum = z^2^* p * q * DEFF/d^2^

utilized with the assumption of:

Z: The Z-score at a 95% confidence interval which is 1.96.

DEFF: the design effect of 2 (assuming an intra-cluster correlation of 1/6).

d: the tolerable margin of error, which is 5%.

Therefore, the sample size was: 1.962 *0.22 *(1–0.22) * 2/0.052 = 528

#### Sampling strategy

**Fig 1:**
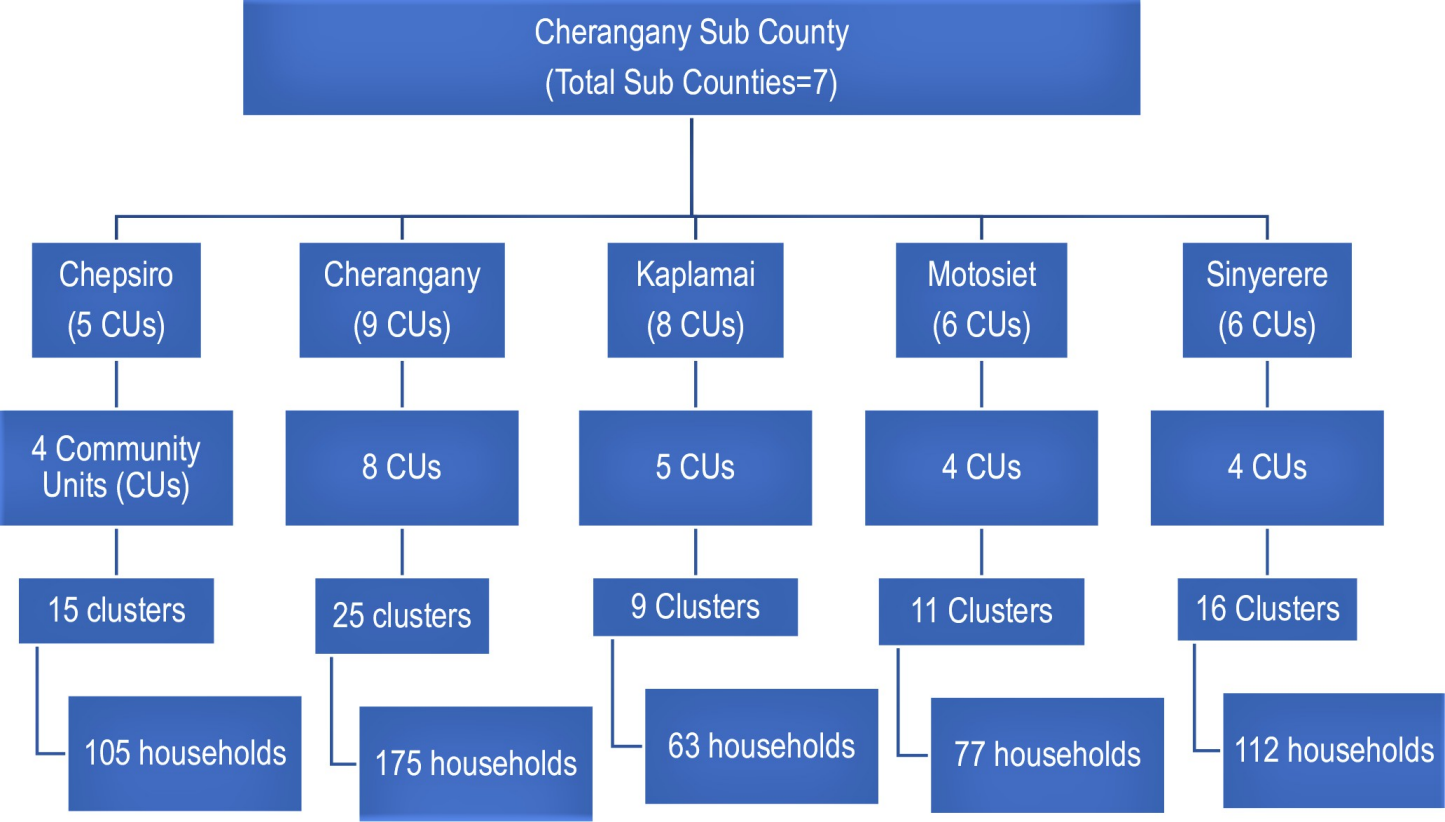
Schematic presentation of the sampling strategy.

A multi-stage cluster sampling strategy was used based on the WHO cluster-based survey method [5]. The wards formed the primary sampling unit, while the community units and villages formed the secondary sampling units. We used simple random sampling using excel to select five wards. The same method was used to determine 25 Community Units (CUs) within the selected wards. The villages within the selected CUs formed the clusters, and 76 clusters (Number of clusters = Sample size/target respondents per cluster = 528/7 = 76 clusters) were sampled with probability proportional to their size according to the estimated number of target households’ data per village provided by the Sub County health authority. The household formed the sample population. The number of households with an eligible child to be selected per cluster was seven. Simple random sampling was used to select the households by spinning a pen at the geographic Centre of the cluster; the same procedure was used to select the subsequent household with an eligible child for the study. If the selected household did not have an eligible child, the same procedure was repeated for the direction of the next household, following the principle of the nearest household with an eligible child. The youngest eligible child was selected for the household with more than one eligible child for coverage (Fig 1).

### Data collection

We used a pretested structured questionnaire to collect data at the household level. We collected socio-demographic, socioeconomic, maternal factors, institutional-related factors, and child vaccination status data. Vaccination information was collected from the caregivers’ vaccination cards shown to the interviewers and from caregivers’ verbal reports on the history of vaccination if the card could not be found.

We classified the child as either fully vaccinated or not fully vaccinated for MCV, which we further classified based on the source of information: vaccination card/ Mother-Child Health (MCH) booklet present or caregiver’s report.

### Operational definitions

#### Measles fully vaccinated

A child who received the two routine vaccination doses of MCV by the time of the survey.

#### Measles partially vaccinated

A child who had received only one dose of MCV routine vaccination at the time of the survey.

#### Measles not vaccinated

A child who had received none of the MCV routine vaccination doses.

#### Not fully vaccinated

A child with either measles partially or not vaccinated.

### Data processing and analysis

The collected data were downloaded into Microsoft Excel^®^ (Seattle). A series of data validation and data checks were created for every variable in the dataset. Data were analyzed using Epi info version 7 [6]. The socio-demographic, maternal, and institutional factors were the independent variables measured for their association with the child’s vaccination status. The child’s vaccination status was the dependent variable categorized as either fully vaccinated or not fully vaccinated. We summarized continuous variables using central tendency and dispersion measures, while categorical variables were summarized using proportions. We conducted bivariate and multivariate analyses to identify independent factors associated with the non-vaccination of MCV. We subjected all variables with a p-value of <0.2 at the bivariate level to logistic regression using the stepwise logistic regression method [7]. The Prevalence Odds ratio (POR) and adjusted POR (aPOR) with its corresponding 95% confidence interval were determined and used to measure the strength of association. The statistical significance was declared at a p-value of less than 0.05.

### Ethical consideration

We sought ethical clearance from the Moi University Institutional Research and Ethics Committee (IREC), the IREC approval number 003846. Permission to conduct the study was then sought from the Trans Nzoia County health department before proceeding to the study site. We sought written consent from the caregivers of the study participants before administering the questionnaire. We administered the questionnaire to those who consented. We also sought assent from the Caregiver if she was <18 years old and permission from her parents, only those who assented were interviewed. We maintained the confidentiality of the individual results by using unique identifiers and a password key.

## Results

### Child and Caregiver’s socio-demographic characteristics

We recruited a total of 536 eligible children. The median age of the participants was 39 months (Interquartile Range 31–50). The female gender was 52.8% (n = 283). The median age of the Caregiver was 30 years (IQR 25–35), the mother as Caregiver contributed the highest proportion of 83.6% (n = 448), and 50.6% (271) had primary level education (Table 1).

**Table 1:** Table showing association of demographic factors of study participants in Cherangany Sub County, Kenya, 2021 (N = 536).

Characteristic	Number	Percent (%)
**Sex (Child)**		
Female	283	52.8
Male	253	47.2
**Age Group (months)**		
24–35	215	40.1
36–47	157	29.3
48–59	162	30.2
Missing data	2	0.4
**Birth Order**		
1–5	488	91.0
>5	48	9.0
**Received other vaccines in the routine vaccination schedule**		
Yes	516	96.3
No	18	3.4
Missing Data	2	0.2
**Family monthly income (Kenya Shillings)**		
<1,000	142	29.2
1,000–10,000	302	62.0
>10,000	43	8.6
**Caregivers’ Age group (years)**		
<18	5	0.9
18–35	403	75.2
36–49	98	18.3
≥50	29	5.4
**Relationship to child (Caregiver)**		
Mother	448	83.6
Father	25	4.7
Other	63	11.8
**Employment status (Caregiver)**		
Self-employed	200	37.3
Housewife	180	33.6
Unemployed	127	23.7
Employed	29	5.4
**Education level (Caregiver)**		
Primary	271	50.6
Secondary	201	37.5
Tertiary	51	9.5
None	13	2.4

### MCV coverage

**Fig 2:**
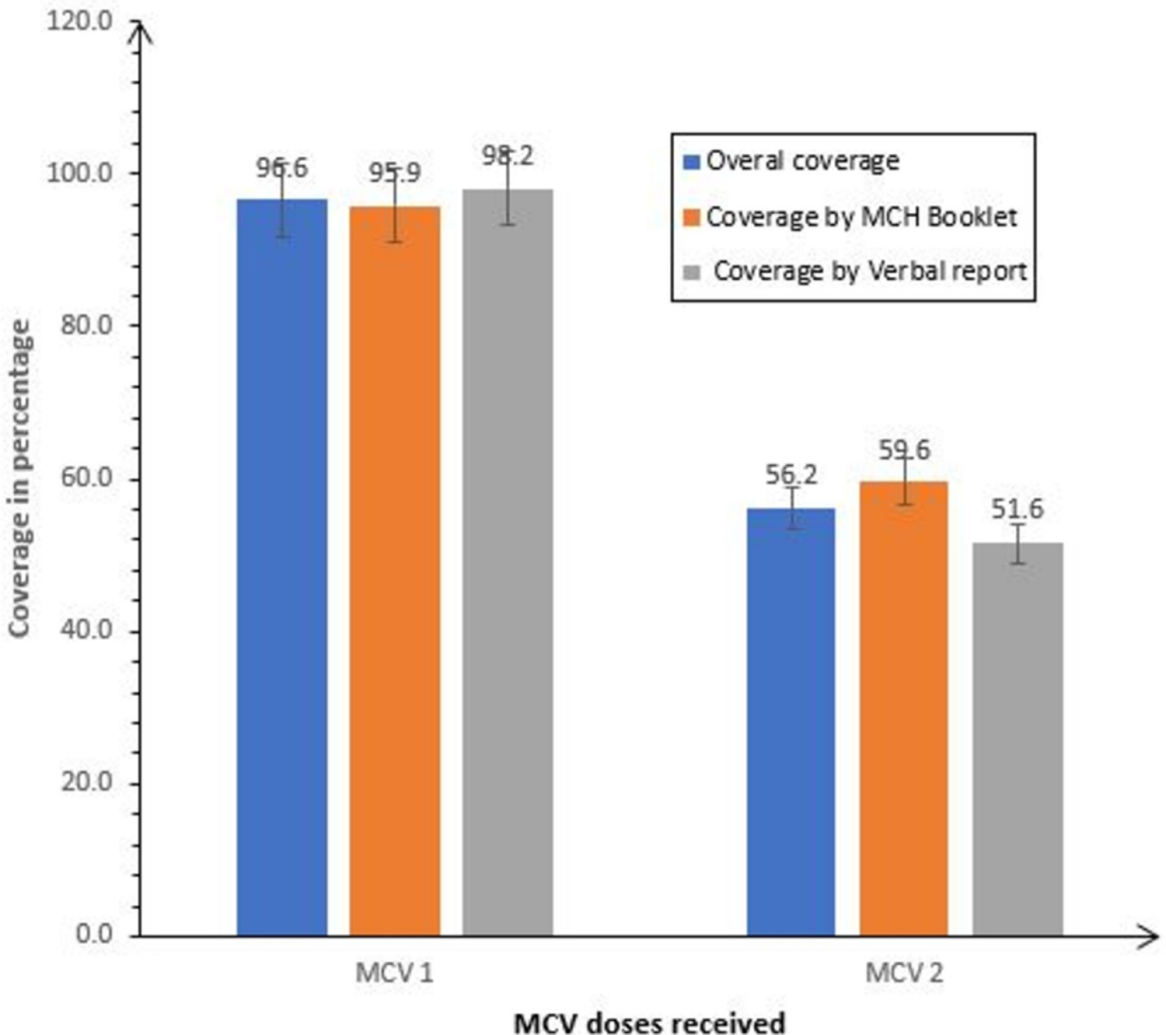
The MCV coverage among the study participants in Cherangany Sub County, 2021. Overall coverage, Coverage by Mother Child (MCH) Booklet, and Coverage by Verbal Report.

The overall MCV dose one (MCV 1) coverage was 96.6% (518/536), and 56.6% (301/536) for MCV dose two (MCV 2). The coverage by MCH booklet was 95.9% (304/317) for MCV 1and 59.6% for MCV 2, while by verbal report was 98.2% (213/217) for MCV 1 and 51.6% for MCV 2 (Fig 2).

### Factors associated with the MCV uptake

The child-related factors significantly associated with MCV uptake at the bivariate level were family monthly income (POR 2.32, 95%CI 1.14–4.72) and a child vaccination status for other scheduled vaccines (POR 0.21, 95% CI 0.07–0.66) (Table 2). The Caregiver’s level of education (POR = 1.82, CI 1.29–2.57), knowledge of the vaccine-preventable diseases (VPDs) (POR = 0.55, 95%CI 0.38–0.80), and knowledge of the number of MCV scheduled doses (POR = 0.13, 95% CI 0.09–0.02) were Caregiver’s factors significantly associated with MCV uptake at the bivariate level (Table 3). None of the institutional factors was significantly associated with the MCV uptake (Table 4). The Caregiver’s knowledge on the number of MCV scheduled doses (POR = 5.7, 95% CI 3.48–9.45) and children whose birth order was ≤5th born (POR = 0.46, 95% CI 0.22–0.95) were significantly associated with MCV uptake at the multivariate analysis (Table 5).

**Table 2:** Table showing association of the child-related factors with MCV uptake in Cherangany Sub County, Kenya, 2021 (N = 536).

Variable	Not fully vaccinated N (Column %)	Fully vaccinated N (Column %)	*POR (95%Confidence Interval)	p-value
**Gender**				
Female	113 (48.09)	170 (56.48)	0.71 (0.51–1.01)	0.06
Male	122 (51.91)	131 (43.52)		
**Age**				
<36 months	80 (37.04)	117 (40.63)	0.86(0.60–1.24)	0.21
≥36 months	136 (62.96)	171 (59.37)		
**Child’s birth order**				
1–5	208(88.51)	280(93.02)	0.58(0.32–1.01)	0.09
> 5	27(11.49)	21(6.98)		
**Family monthly income (Kenya Shillings)**				
<10,000	197(94.71)	247(88.53)	2.32(1.14–4.72)	0.02
≥10,000	11(5.29)	32(11.47)		
**Immunization status for other Vaccines**				
Fully	221(94.04)	295(98.66)	0.21(0.07–0.66)	0.01
Partially	14(5.96)	4(1.34)		

*POR Prevalence Odds Ratio

**Table 3:** Caregiver’s factors associated with Measles containing vaccine uptake, Cherangany Sub- County, 2021 (N = 536).

Variable	Not fully vaccinated N (Column %)	Fully vaccinated N (Column %)	*POR (95% Confidence Interval)	p-value
**Age group of Caregiver**				
≥45	23(9.79)	25(8.33)	1.19(0.66–2.16)	0.56
<45	212(90.21)	275(91.67)		
**Caregiver’s level of education**				
<High school	144(61.28)	140(46.51)	1.82 (1.29–2.57)	<0.001
≥High school	91(38.72)	161(53.49)		
**Decision maker**				
Father	13(5.53)	16(5.32)	1.04(0.49–2.21)	1
Anyone	222(94.47)	285(94.68)		
**Caregiver’s Employment status**				
Unemployed	135(57.45)	172(57.14)	1.01(0.72–1.43)	0.94
Employed	100(42.55)	129(42.86)		
**Number of Antenatal visits**				
≥4	110(40.74)	160(56.34)	0.85(0.60–1.22)	0.38
<4	100(47.62)	124(43.66)		
**Place of delivery**				
No SBA	60(25.53)	58(19.27)	1.44(0.95–2.16)	0.08
SBA	175(74.47)	243(80.73)		
**Knowledge of Vaccine-Preventable Diseases**				
Yes	141(60)	220(73.09)	0.55(0.38–0.80)	<0.001
No	94(40)	81(26.91)		
**Knowledge of Measles Containing Vaccine scheduled doses**				
Yes	32(13.62)	164(54.49)	0.13(0.09–0.02)	<0.001
No	203(86.38)	137(45.51)		

*POR Prevalence Odds Ratio

**Table 4:** Institutional factors associated with Measles vaccine uptake, Cherangany Sub County, 2021 (N = 536).

Variable	Not fully vaccinated N (Column %)	Fully vaccinated N (Column %)	*POR (95% Confidence Interval)	p-value
**Distance to the Health Facility (Km)**				
≤5	191(81.28)	250(83.06)	0.89(0.57–1.38)	0.59
>5	44(18.72)	51(16.94)		
**Visited Health Facility and not vaccinated**				
Yes	76(32.34)	87(28.9)	1.18(0.81–1.70)	0.39
No	159(67.66)	214(71.1)		
**Satisfaction with Routine Immunization services**				
Yes	214(91.45)	270(90.3)	1.14(0.63–2.09)	0.65
No	20(8.55)	29(9.7)		

*POR Prevalence Odds Ratio

**Table 5:** Table showing results of the multivariate analysis of factors associated with MCV uptake in Cherangany Sub County.

Variable	*aPOR	95%Confidence Interval	p-Value
Vaccination against other vaccines (Fully/Partially)	2.22	0.68–7.26	0.19
Knowledge of Vaccine-Preventable Diseases (Yes/No)	1.42	0.91–2.20	0.12
Knowledge Measles Containing Vaccine scheduled doses (Yes/No)	5.73	3.48–9.45	< 0.001
Family monthly income (Kenya Shillings) (<10,000/≥10,000)	1.29	0.52–3.17	0.59
Caregiver’s Education level (<High school/≥High school	1.14	0.74–1.77	0.56
Child’s gender (Male/Female)	0.85	0.56–1.29	0.44
Birth order (1-5/>5^th^ born)	0.46	0.22–0.95	0.04

*aPOR Prevalence Odds Ratio

## Discussion

We sought to determine the coverage of MCV 1 and 2 doses and factors associated with its uptake in Cherangany Sub County, Trans Nzoia County, Kenya. Our study showed a significant drop in the coverage of MCV 2 compared to MCV 1. The factors significantly and independently associated with the uptake were; knowledge of the number of scheduled MCV doses and the child’s birth order.

Most of the children had received MCV 1 compared to those who had received two doses. This study’s MCV 1 and 2 coverage was high compared to a study that reviewed vaccination data between 2003 and 2016 in Kenya, showing an MCV 1 coverage between 65% - 86% and MCV 2 coverage of below 50% [8]. It is also high compared to studies done in Ethiopia [9]. This improvement is likely a result of innovations in implementing routine vaccination programs in Kenya [10]. This may also result from the Sub County carrying out outreach vaccination activities in coordination with the local community for the areas disadvantaged by topography and increased distance to the health facilities. The significant dropout rate from MCV1 and 2 may be due to the long duration between the first dose given at nine months and the second dose at 18 months which is nine months apart; thus, the Caregiver is likely to forget, as seen in similar studies in Nigeria [11]. Other reasons for the dropout include negligence, logistical problems, and a low level of mothers’ knowledge towards vaccination and its schedule, as reported in Ethiopia [12]. The MCV 2 coverage is way below the WHO recommended coverage of ≥95% for the two MCV doses to eliminate measles [13]. This low coverage also shows that the Sub County is still at risk of measles outbreaks because the >95% MCV 1 coverage alone does not confer population immunity. Therefore, there is a need for MCV supplemental vaccination activities such as Vaccination campaigns to help improve population immunity. The MCV 2 coverage can also be improved by establishing or strengthening the defaulter tracing system. The children who receive the first dose but fail to turn up for the second dose as scheduled are followed up and referred for vaccination.

Children who had received all the other vaccines were more likely to be fully vaccinated with MCV. These results are similar to Noh et al. where the Caregiver’s prior contact with the health care facilities was a proxy for subsequent complete child vaccination status because apart from the health-seeking behavior, it was a signal for healthcare facility accessibility [14]. This is possible because the Caregiver frequently interacts with the health care providers who provide reminders on the importance of the vaccine and subsequent vaccination schedule hence the likelihood of adherence to the schedule. The family monthly income was also significantly associated with MCV uptake in this study. These results were consistent with studies done in Nigeria and Pakistan [14–16]. The monthly income can be used to estimate the family’s wealth status. It determines the amount that can be used for the indirect costs of vaccination, such as transport to the vaccination post, which influences the health-seeking behavior and, in turn, the coverage. There is need for the use of mobile clinics and outreaches targeting vulnerable mothers and infants to increase access to services with reduced indirect costs in the general population.

Children whose caregivers had secondary education and higher were more likely to be Measles fully vaccinated than those who had a primary level or no education. This was consistent with studies done in various parts of the world, showing that maternal education is associated with vaccine uptake [11, 17–29]. In addition, several studies have shown that caregiver education is an empowerment route that leads to greater receptivity to information concerning public health aimed at boosting child vaccination rates [14, 19, 29–32]. This empowerment tends to improve health-seeking behavior through changing attitudes, traditions, and beliefs, increasing autonomy, and decision making. Education also helps build social networks that provide good health behavior-related information and find available healthcare services.

Knowledge of VPDs was significantly associated with MCV uptake. Knowledge of VPDs was significantly associated with MCV uptake. This may be because caregivers are more likely to make informed decisions when given valid information, similar to a study done in China [28]. In contrast, knowledge of VPDs was not enough to improve uptake; it needs to be coupled with positive attitudes towards vaccination and a good perception of vaccination services in a study done in Uganda [33]. Moreover, the same study showed that though most women identified polio and measles as VPD, the under-vaccinated children did not receive the last doses of the two diseases [33]. Therefore, the uptake of specific vaccines is likely not wholly dependent on the evidence of knowledge of VPDs but may be influenced more by the general vaccination program, including benefits, the number of doses, schedule, and logistics. Lack of knowledge of the number of MCV doses was a risk factor for not being fully vaccinated. Knowledge of the various operational aspects of service such as schedule, number of doses, rescheduling, and options in case of a missed appointment or lost vaccination card help ensure complete vaccination. On the other hand, a cohort study in the same country in a different area found that mothers who did not know the benefits of vaccination were reluctant to follow health care workers’ instructions on vaccine schedules [34]. This is a likely case because awareness of the benefits of vaccination helps the Caregiver to be confident and have trust in what is administered to their children.

We found that the child’s birth order was significantly associated with MCV uptake at logistic regression. These results are consistent with studies done in Ethiopia, Cameroon, and India [34–36]. This is possibly due to parents having developed confidence in their child’s healthcare because of years of experience from previous children and could dismiss the importance of vaccination. It could also be that the other children experienced an adverse reaction to vaccination, leading the parents to believe that vaccination was risky. In addition, the many children may lead to the diversion of attention due to an increased number of duties, as found in a study in Cameroon [35]. The high birth order may also increase the demand for the Caregiver’s limited resources and time. There may be a need to encourage these mothers to consider family planning to the number of children they can comfortably take care of, considering the available resources.

### Limitations

This study included the report on vaccination status by the presence of the MCH booklet/vaccination card and verbal report by the Caregiver, which may have introduced recall bias. However, there was a slight difference in the coverage for the two reports. The data on the vaccination status and associated factors were collected at the same time; thus, it might not be possible to explain whether the factors preceded/influenced the vaccination status.

## Conclusion

The MCV 2 coverage was way below the WHO recommended coverage of ≥95%. The lack of knowledge of the number of MCV scheduled doses and the high birth order are the main risk factors independently associated with a child not being fully vaccinated with the MCV in Cherangany Sub County. There is a need to strengthen the defaulter tracing system to follow up the children who default after receiving MCV 1 coupled with interventional studies focusing on the identified factors to determine their impact on the MCV vaccine uptake.

## Supporting information

S1 Data (XLSX)
